# Midline invasion predicts poor prognosis in diffuse hemispheric glioma, H3 G34-mutant: an individual participant data review

**DOI:** 10.1007/s11060-024-04587-5

**Published:** 2024-03-01

**Authors:** Yasuhito Kegoya, Yoshihiro Otani, Yohei Inoue, Ryo Mizuta, Fumiyo Higaki, Kana Washio, Shinichiro Koizumi, Kazuhiko Kurozumi, Joji Ishida, Kentaro Fujii, Norio Yamamoto, Yoshihiro Tanaka, Isao Date

**Affiliations:** 1 Department of Neurological Surgery, Okayama University Graduate School of Medicine, Dentistry, and Pharmaceutical Sciences, 2-5-1, Shikata-cho, Kita-ku, 700-8558 Okayama-shi, Okayama, Japan; 2 Department of Radiology, Okayama University Faculty of Medicine, Dentistry and Pharmaceutical Sciences, 2-5-1, Shikata-cho, Kita-ku, 700-8558 Okayama-shi, Okayama, Japan; 3 Department of Pediatrics, Okayama University Graduate School of Medicine, Dentistry, and Pharmaceutical Sciences, 2-5-1, Shikata-cho, Kita-ku, 700-8558 Okayama-shi, Okayama, Japan; 4 Department of Neurosurgery, Hamamatsu University School of Medicine, 1-20-1, Handayama, Chuo-ku, 431-3192 Hamamatsu-shi, Shizuoka, Japan; 5 Department of Epidemiology, Okayama University Graduate School of Medicine, Dentistry, and Pharmaceutical Sciences, 2-5-1, Shikata-cho, Kita-ku, 700-8558 Okayama-Shi, Okayama, Japan; 6 Division of Epidemiology, Graduate School of Public Health, Shizuoka Graduate University of Public Health, 4-27-2, Kitaandou, Aoi-ku, 420-0881 Shizuoka-Shi, Shizuoka, Japan

**Keywords:** Diffuse hemispheric gliomas, H3 G34-mutation, Midline invasion, Frontal lobe, Gross total resection

## Abstract

**Introduction:**

Diffuse hemispheric glioma, H3 G34-mutant (DHGs), is a newly categorized tumor in pediatric-type diffuse high-grade gliomas, World Health Organization grade 4, with a poor prognosis. Although prognostic factors associated with genetic abnormalities have been reported, few reports have examined the clinical presentation of DHGs, especially from the viewpoint of imaging findings. In this study, we investigated the relationship between clinical factors, including imaging findings, and prognosis in patients with DHGs.

**Methods:**

We searched Medline through the PubMed database using two search terms: “G34” and “glioma”, between 1 April 2012 and 1 July 2023. We retrieved articles that described imaging findings and overall survival (OS), and added one DHG case from our institution. We defined midline invasion (MI) as invasion to the contralateral cerebrum, brainstem, corpus callosum, thalamus, and basal ganglia on magnetic resonance imaging. The primary outcome was 12-month survival, estimated using Kaplan–Meier curves and logistic regression.

**Results:**

A total of 96 patients were included in this study. The median age was 22 years, and the proportion of male patients was 48.4%. Lesions were most frequently located in the frontal lobe (52.6%). MI was positive in 39.6% of all patients. The median OS was 14.4 months. Univariate logistic regression analysis revealed that OS was significantly worse in the MI-positive group compared with the MI-negative group. Multivariate logistic regression analysis revealed that MI was an independent prognostic factor in DHGs.

**Conclusions:**

In this study, MI-positive cases had a worse prognosis compared with MI-negative cases.

**Previous presentations:**

No portion of this study has been presented or published previously.

**Supplementary Information:**

The online version contains supplementary material available at 10.1007/s11060-024-04587-5.

## Introduction

Diffuse hemispheric gliomas, H3 G34-mutant (DHGs), are a newly categorized tumor in pediatric-type diffuse high-grade gliomas in the 2021 World Health Organization classification, with a poor prognosis [1, 2]. DHGs are caused by an amino acid substitution in the histone gene *H3F3A* (H3.3) in which glycine at position 34 is replaced by arginine or rarely valine (G34R/V) [3–5]. Recent studies have shown that the majority of DHGs have *ATRX* and *TP53* alterations, and approximately half have *PDGFRA* abnormalities [6]. Additionally, most have *MGMT* promotor methylation [4, 7]. These tumors are most commonly located in the cerebral hemispheres, particularly in the frontoparietal lobes [8]. Histologically, the majority present as high-grade gliomas, such as glioblastomas (GBMs) or anaplastic astrocytomas, while others present as primitive neuroectodermal tumors or low-grade gliomas, pathologically [6, 9–11].

Because DHGs are reported to occur in less than 1% of all gliomas, previous studies have involved small sample sizes. Thus, prognostic factors have not been well investigated; however, the prognosis of DHGs is dismal. Recently, Crowell et al. performed a systematic review of 135 patients with DHGs, and univariate analysis showed that age and degree of resection were significantly associated with overall survival (OS) [6]. Other researchers showed that G34V-mutant tumors had significantly worse OS compared with G34R-mutant tumors [12]. These studies evaluated the prognostic impact of clinical factors and genetic alterations, but the impact of radiographic features has not been well investigated. Only one report showed that ill-defined DHG margins were associated with a worse prognosis compared with well-defined margins [8]. Therefore, the purpose of the present study was to clarify the relationship between imaging findings and prognosis in patients with DHGs.

## Methods

This study was a non-registered individual participant data review. No protocol was prepared.

### Patient cohort

**Fig. 1:**
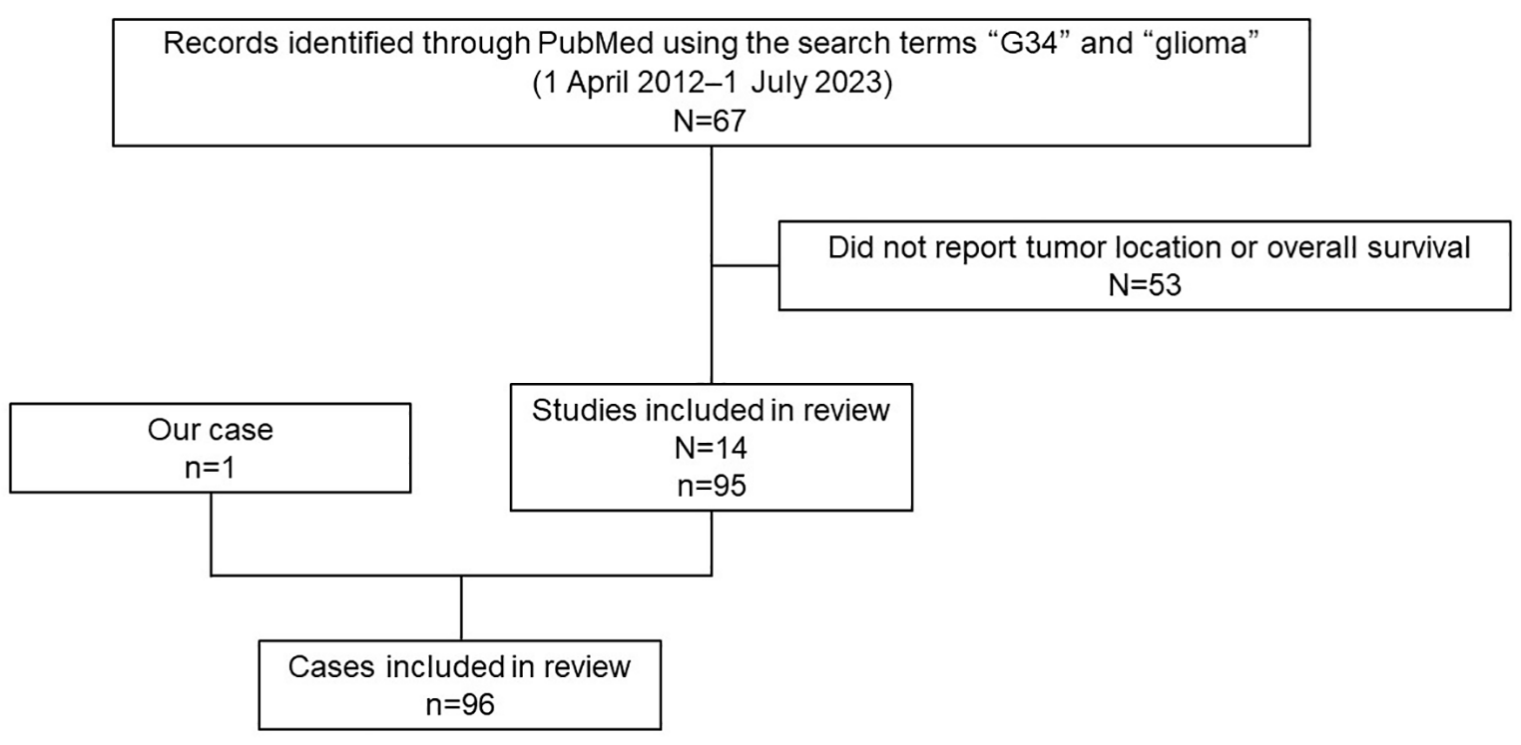
Flowchart demonstrating the case selection process in this study. N: number of studies, n: number of cases

We searched Medline through the PubMed database using two search terms, “G34” and “glioma”, between 1 April 2012 and 1 July 2023. In the present cohort, two neurosurgeons independently conducted the search and selected the cases in August 2023. Among the identified studies, we excluded studies that did not report the tumor location or OS. Studies containing cases duplicated in different published articles were also excluded. There were no restrictions on race or age (Fig. 1).

Additionally, after obtaining institutional review board approval (1608-026) from Okayama University Hospital, we retrospectively reviewed our medical records for cases. We performed immunohistochemistry for patients diagnosed as having glioma in our institute between January 2000 and May 2022 using rabbit monoclonal recombinant antibodies to histone H3.3 G34R or G34V (RevMAb BioSciences, San Francisco, CA, USA; 1:250 dilution for G34R and G34V).

### Data extraction

Data were extracted from all available sources in the selected manuscripts and our medical records. Patient demographics and radiographic characteristics comprised age, sex, lesion location, and imaging findings (contrast enhancement, necrosis, cyst formation, apparent diffusion coefficient (ADC) value). Molecular findings, such as H3.3 G34, MGMT, *ATRX* status and patient outcomes, and surgical details, were also collected.

Midline invasion (MI) was defined as positive if lesions involved the contralateral cerebrum, brainstem, corpus callosum, thalamus, or basal ganglia on either non-contrast-enhanced or contrast-enhanced magnetic resonance imaging. To determine midline invasion, we reviewed only the initial upfront MRI to avoid post-treatment changes. Although ADC values would be valuable to differentiate edema from tumor infiltration, only 25 of 96 patients have the data of ADC values. Therefore we could not use the ADC value secondarily to determine the presence of hypercellular tumor with midline invasion or edema crossing the midline.

Patients were followed from the date of the diagnosis until the date of death or the end of the study period, whichever occurred first. In the present study, patients who were alive at the end of the study were censored only because no patients were lost to follow-up. The primary outcome was defined as OS at 12 months.

### Statistical analysis

Continuous variables were expressed as median (interquartile range), and categorical variables were expressed as n (%). We used a t-test to compare the continuous variables of two samples and used the chi-square test to compare categorical variables. Survival probabilities were estimated using the Kaplan–Meier method, and the log-rank test was used to compare the survival distributions between groups, as follows: age (≤ 19 years, 20–29 years, and ≥ 30 years), sex, laterality (right, left, bilateral), MI (positive, negative), G34 status (G34R-positive, G34V-positive), *MGMT* promoter status (unmethylated, methylated), contrast enhancement (positive, negative), and extent of resection (biopsy, subtotal resection, gross total resection (GTR)). Additionally, using a logistic regression model, we calculated odds ratios (ORs) and 95% confidence intervals (CIs) for each item using 12-month survival as the outcome. The variables in the multivariate logistic regression model comprised age, sex, MI, and G34 status on the basis of clinical knowledge and previous reports [12]. Extent of resection was excluded owing to its strong correlation with MI (multicollinearity). *p* < 0.05 was considered statistically significant, and all analyses were performed using GraphPad Prism (version 9.00 for Windows; GraphPad Software, La Jolla, CA, USA).

## Results

**Fig. 2:**
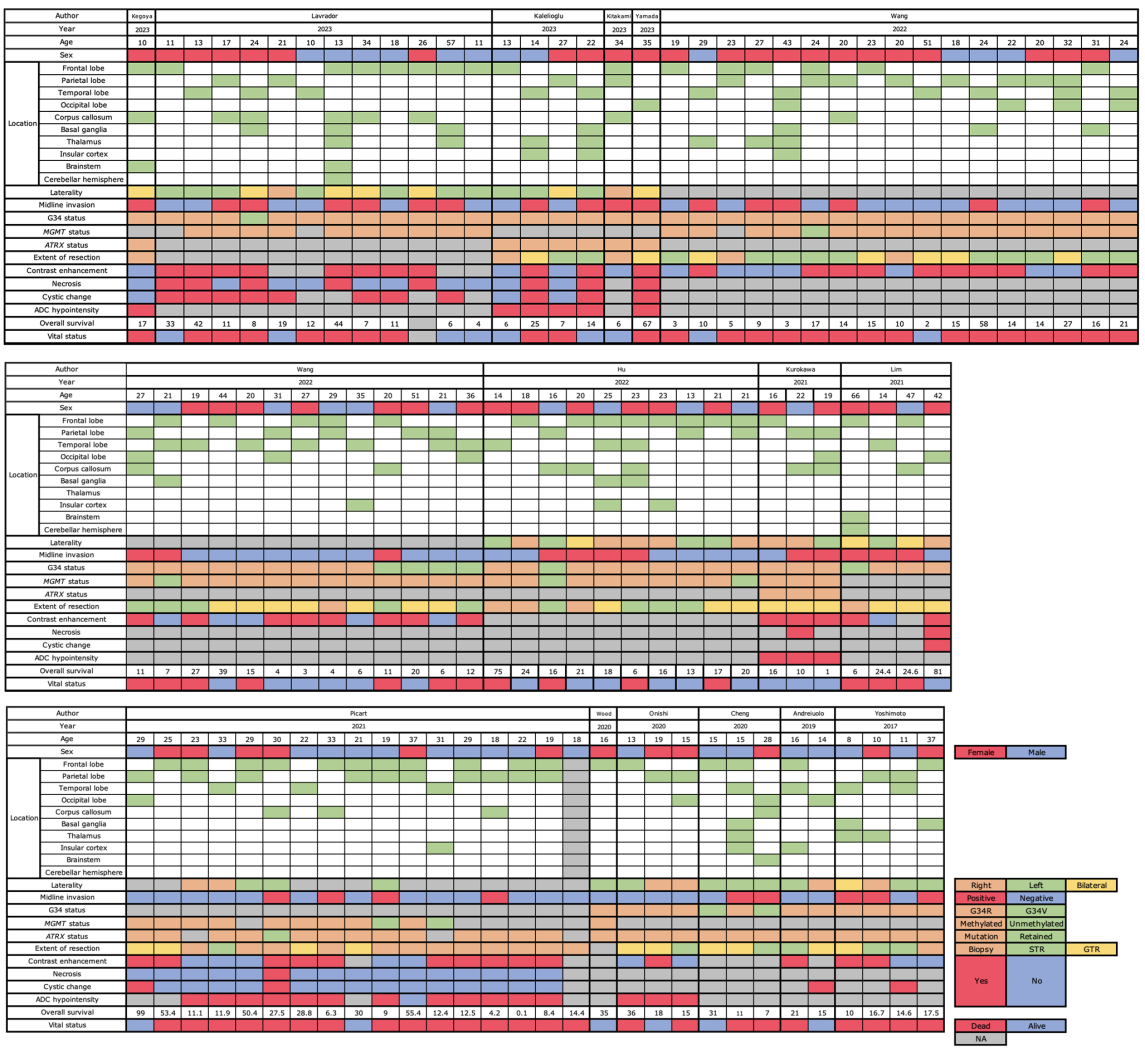
Summary of the clinical, radiographic, and genetic features in patients with diffuse hemispheric gliomas, H3 G34-mutant cases. ADC: apparent diffusion coefficient

Of the 67 identified studies, 95 cases in 14 articles [7, 8, 13–24] met the inclusion criteria of reporting the tumor location and OS. Additionally, we included one patient from our institute whose diagnosis was confirmed by both immunohistochemistry and genome-wide DNA methylation profiling (Table 1; Fig. 2, Supplementary Fig. 1). The patients’ demographic data are summarized in Table 1. Forty-three of the 96 patients (44.8%) were male, and the median age was 22 years (whole range, 8–66 years) (interquartile range, 18–29), which was older than previous reports [6]. The data except for age, sex, and MI included missing data (Table 1). The neurological findings were shown in supplementary Table 1.

**Table 1:** Clinical characteristics of the patients on the basis of 12-month overall survival

Variable	Overall (n = 96)	Death(−) (n = 57)	Death(+) (n = 26)	*P* value
**Age**				
Median age at diagnosis [year, IQR]	22 [18–29]	21[15–29]	22.5[19–27]	0.392
**Sex**				
** Male**	43 (44.8%)	23 (40.4%)	12 (46.2%)	0.64
Female				
**Laterality**				
Right	17 (31.5%)	11 (32.4%)	4 (30.8%)	1
Left	27 (50.0%)	18 (52.9%)	5 (38.5%)	0.517
Bilateral	10 (18.5%)	5 (14.7%)	4 (30.8%)	0.237
Number of patients with missing data	42	23	13	
**Location**				
Frontal lobe	50 (52.6%)	27 (48.2%)	15 (57.7%)	0.482
Parietal lobe	38 (40.0%)	21 (37.5%)	10 (38.5%)	1
Temporal lobe	30 (31.6%)	17 (30.4%)	9 (34.6%)	0.8
Occipital lobe	14 (14.7%)	9 (16.1%)	3 (11.5%)	0.744
Corpus callosum	18 (18.9%)	7 (12.5%)	8 (30.8%)	0.066
Basal ganglia	13 (13.7%)	6 (10.7%)	6 (23.1%)	0.182
Thalamus	10 (10.5%)	4 (7.1%)	4 (15.4%)	0.256
Insular cortex	11 (11.6%)	7 (12.5%)	3 (11.5%)	1
Brain stem	4 (4.2%)	2 (3.6%)	2 (7.7%)	0.588
Cerebellum	2 (2.1%)	1 (1.8%)	1 (3.8%)	0.536
Number of patients with missing data	1	1	0	
**Midline invasion**				
Positive	38 (39.6%)	16 (28.1%)	16 (61.5%)	0.007
Negative				
**Extent of resection**				
Gross total resection	35 (42.2%)	25 (49.0%)	3 (13.6%)	0.004
Subtotal resection	27 (32.5%)	17 (33.3%)	10 (45.5%)	0.429
Biopsy	21 (25.3%)	9 (17.6%)	9 (40.9%)	0.039
Number of patients with missing data	13	6	4	
**Contrast enhancement**				
Yes	48 (62.3%)	28 (70.0%)	12 (52.2%)	0.183
No	29 (37.7%)	12 (30.0%)	11 (47.8%)	
Number of patients with missing data	19	17	3	
**Necrosis**				
Yes	11 (30.6%)	8 (42.1%)	1 (8.3%)	0.101
No	25 (69.4%)	11 (57.9%)	11 (91.7%)	
Number of patients with missing data	60	38	14	
**Cyst formation**				
Yes	16 (48.5%)	11 (57.9%)	4 (33.3%)	0.273
No	17 (51.5%)	8 (42.1%)	8 (66.7%)	
Number of patients with missing data	63	38	14	
**ADC hypointensity**				
Yes	24 (96.0%)	13 (92.9%)	8 (100%)	1
No	1 (4.0%)	1 (7.1%)	0 (0%)	
Number of patients with missing data	71	43	18	
**H3F3A alteration**				
G34 R	70 (88.6%)	43 (91.5%)	14 (73.7%)	0.106
G34 V	9 (11.4%)	4 (8.5%)	5 (26.3%)	
Number of patients with missing data	17	10	7	
***MGMT*** **promoter methylation**				
Yes	57 (89.1%)	32 (88.9%)	15 (88.2%)	1
No	7 (10.9%)	4 (11.1%)	2 (11.8%)	
Number of patients with missing data	32	21	9	
***ATRX*** **alteration**				
Yes	36 (97.3%)	22 (95.7%)	10 (100%)	1
No	1 (2.7%)	1 (4.3%)	0 (0%)	
Number of patients with missing data	59	34	16	
**Patient survival**				
Median overall survival [months, IQR]	14.4 [8.2–22.5]	20 [15–31.0]	7.5 [6–10.8]	< 0.001

ADC: apparent diffusion coefficient, IQR: interquartile range

### Radiographic findings

An ipsilateral lesion was observed in 81.5% (44/54) of the patients, and the left hemisphere was slightly dominant (27/54 cases, 50.0%). Bilateral lesions were observed in 18.5% (10/54) of the patients. Consistent with previous reports [6, 18], tumors were located most often in the frontal lobe (50/95cases, 52.6%). Contrast enhancement was seen in 62.3% (48/77) of the patients, necrosis in 30.6% (11/36), and cyst formation in 48.5% (16/33). A low ADC value was observed in 96.0% (24/25) of the cases. MI was observed in 39.6% (38/96) of the cases, namely 18 in the corpus callosum, 13 in the basal ganglia, 10 in the contralateral cerebrum, 10 in the thalamus, and 4 in the brainstem (some patients had lesions in more than one location).

### Genetic alterations

Regarding the missense mutation in the *H3-3 A* gene, p.G34R was observed in 88.6% (70/79) of the cases, whereas p.G34V was observed in 11.4% (9/79). Additionally, 97.3% (36/37) of the cases had alterations in the *ATRX* gene. *MGMT* promoter methylation was identified in 89.1% (57/64) of the cases.

### Outcomes

Of the 83 patients who underwent surgery, biopsy results were reported in 25.3% (21/83) of the cases; subtotal resection results were reported in 32.5% (27/83), and GTR results were reported in 42.2% (35/83) (Table 1). The median OS of all cases was 14.4 months, and the 12- and 24-month survival rates were 68.1% and 42.0%, respectively.

### MI predicts the prognosis of DHGs, H3 G34-mutant

**Fig. 3:**
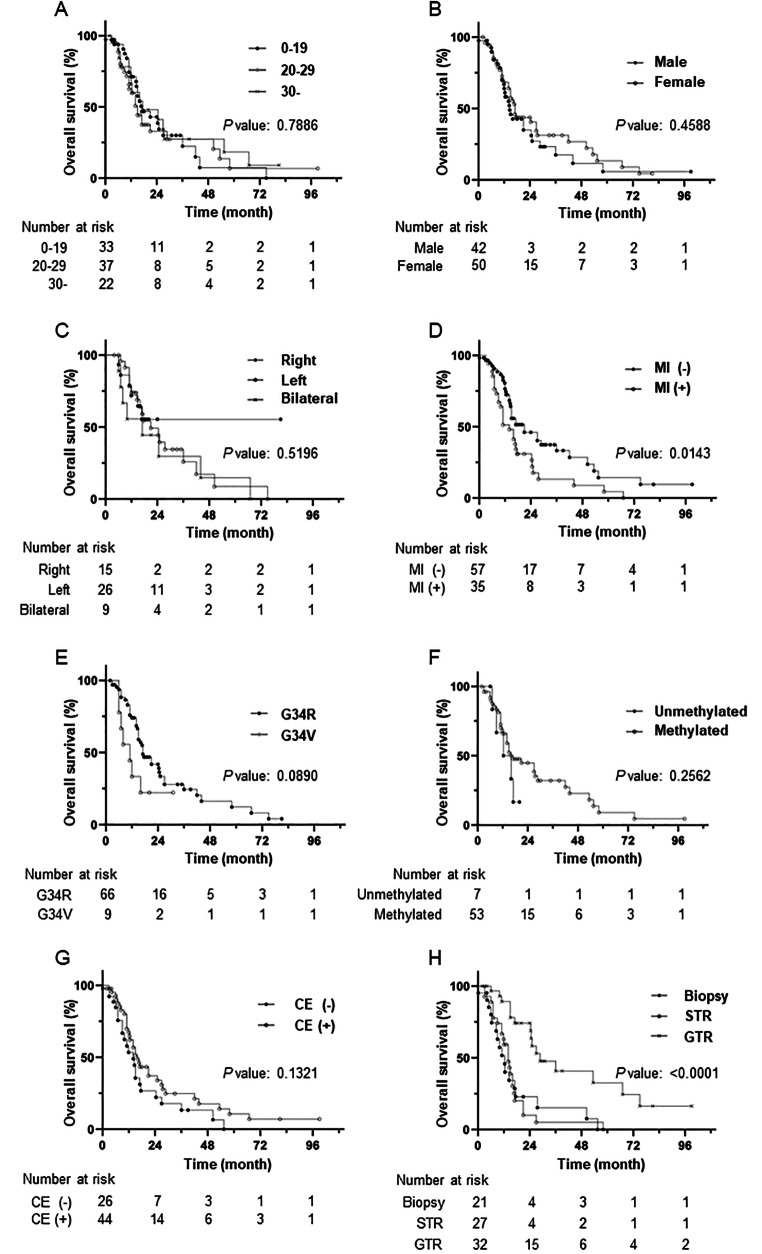
Kaplan–Meier survival curve demonstrating overall survival. Overall survival was evaluated with (A) age, (B) sex, (C) laterality, (D) midline invasion (MI), (E) genetic alteration in H3F3A, (F) methylation status of the MGMTMGMT promoter, (G) contrast enhancement (CE), and (H) extent of resection at the primary surgery. STR: subtotal resection, GTR: gross total resection

Figure 3 shows the OS curves for each clinical, radiographic, and genetic feature. In the univariate analysis, compared with the MI-negative group, OS was significantly shorter in the MI-positive group (*p* = 0.0135) and significantly longer in the GTR-achieved vs. other two group (*p* < 0.0001).

Univariate logistic regression analysis with 12-month OS as the outcome showed that the MI-positive group had a significantly higher mortality than that in the MI-negative group (OR = 4.48, 95% CI = 1.70–12.4; *p* < 0.01), and the GTR-achieved group had a significantly lower mortality than that in the group that underwent biopsy alone (OR = 0.14, 95% CI = 0.03–0.57; *p* = 0.01) (Table 2).

**Table 2:** Univariate analysis of 12-month overall survival

Characteristic	Odds ratio (95% CI)	*P* value
Age	1.02 (0.98–1.07)	0.32
Sex		
Female	Ref.	
Male	1.27 (0.49–3.24)	0.62
Midline Invasion		
Negative	Ref.	
Positive	4.48 (1.70–12.4)	< 0.01
Laterality		
Left	Ref.	
Right	1.31 (0.27–6.03)	0.73
Bilateral	2.88 (0.54–15.6)	0.21
G34 status		
G34R	Ref.	
G34V	1.00 (0.19–4.27)	> 0.99
*MGMT* promoter methylation		
unmethylated	Ref.	
methylated	0.94 (0.16–7.30)	0.94
Contrast enhancement		
Negative	Ref.	
Positive	0.53 (0.18–1.50)	0.23
Extent of resection		
Biopsy	Ref.	
Subtotal resection	0.65 (0.20–2.16)	0.48
Gross total resection	0.14 (0.03–0.57)	0.01

CI: confidence interval

Furthermore, multivariate logistic regression was performed to examine the association of MI with 12-month OS as the outcome. Compared with the MI-negative group, the MI-positive group had significantly higher mortality after 12 months (OR = 3.60, 95% CI = 1.20–11.5; *p* = 0.02) (Table 3).

**Table 3:** Multivariate analysis of 12-month overall survival

Characteristic	Odds ratio (95% CI)	*P* value
Age	1.02 (0.97–1.08)	0.39
Sex		
Female	Ref.	
Male	1.20 (0.37–3.89)	0.76
Midline Invasion		
Negative	Ref.	
Positive	3.60 (1.20–11.5)	0.02
G34 status		
G34R	Ref.	
G34V	0.63 (0.10–3.20)	0.6

CI: confidence interval

## Discussion

DHGs were a newly defined tumor entity in the World Health Organization classification 5th edition, and were reported to occur in less than 1% of all gliomas [1]. Yoshimoto et al. reported that in their analysis of 411 gliomas, 4 tumors had G34R mutations [13]. In contrast, more than 30% of high-grade gliomas in adolescents and young adults harbor heterozygous mutations in the non-canonical H3.3 variant, resulting in glycine 34 to arginine or valine (G34R/V) amino acid substitution [25]. DHGs exhibit primitive neuroectodermal tumor-like or GBM-like histology and almost invariably carry *ATRX* and *TP53* mutations, and lack immunoreactivity for OLIG2 [18]. Additionally, DHGs frequently exhibit MGMT promoter methylation and lack TERT promoter mutations [18].

Although DHGs were first reported in 2012, precise treatment and prognostic factors have not been well elucidated [3, 4]. Regarding treatment, a systematic review showed that most cases underwent upfront surgical resection; however, GTR was achieved in less than 50% of the cases [6]. Radiotherapy and chemotherapy have also been used as initial treatment, but the details have not been well described. Of the 31 patients in this study with detailed chemotherapy regimen information, 20 (64%) received temozolomide-based therapy [6]. Although the significance of MGMT promoter methylation in DHGs has not been investigated, Crowell et al. showed that patients harboring MGMT promoter methylation showed superior survival [6]. Vuong et al. reported that *PDGFRA* and *EGFR* amplification had a negative prognostic impact, with the G34V genotype having a worse prognosis than that for G34R [12]. In their review, methylation of the MGMT promoter was also observed in most DHGs. Furthermore, G34V-positive DHGs tended to have a worse prognosis than G34R-positive DHGs.

In this study, we investigated the radiographic and genetic factors related to prognosis in patients with DHGs. To the best of our knowledge, this is the largest study to have analyzed the radiographic features of DHGs. Korshunov et al. investigated 81 patients with DHGs and reported that 80% of the tumors were located in the temporal and parietal lobes [26]. In our study, the most common tumor location was the frontal lobe, but many tumors also invaded the parietal and temporal lobes. Additionally, DHGs were characterized by slight gadolinium contrast enhancement, peritumoral edema on T2-weighted/fluid-attenuated inversion recovery imaging, and hyperintensity on diffusion-weighted imaging or a low ADC value [16]. In the present study, 94% of the cases had a low ADC value, which can be a characteristic imaging finding. According to Ohnishi et al., diffusion-weighted imaging hyperintensity and a low ADC value may be associated with high cellularity in DHGs [16].

Previous studies have shown that deep supratentorial extension involving the thalamus, basal ganglia, and corpus callosum in GBMs is associated with a poor prognosis [27]. According to Dayani et al., GBM spread through the corpus callosum to the contralateral cerebrum (“butterfly GBM”) was associated with a worse prognosis than that for localized GBMs, with a median overall survival of 3.2 months [28]. Although radiographic features, such as hemispheric location, are diagnostic criteria for DHGs, the relationship between imaging findings and prognosis has not been well investigated. Only one study revealed that OS for DHGs with ill-defined margins was significantly lower than that for DHGs with well-defined margins [8]. Our study summarized the clinical and radiological findings of 93 DHG cases, and MI was identified as a prognostic factor.

There is no difference in prognosis if GTR can be achieved, even in cases with tumor invasion into deep structures, in patients with GBMs [27]. Because tumor invasion into deep structures interferes with GTR, the addition of adjuvant therapy fails to suppress the growth of residual tumor, resulting in short OS. In the present study, the DHG MI-negative group had better 12-month OS compared with the MI-positive group. However, univariate analysis suggested that the resection rate was strongly correlated with OS. In this study, we analyzed MI as a prognostic factor; however, the surgical technique may be the most important factor because surgical strategies and resection rates are determined on the basis of imaging findings. Thus, safe GTR may prolong OS in MI-negative patients with DHGs. Recently, there have also been reports of using language mapping and 5-aminolevulinic acid during surgery for DHGs [24]. Combining multiple modalities, such as tractography, the use of navigation systems and intraoperative magnetic resonance imaging, and awake surgery, which are usually used in glioblastoma surgery [29], enables safe and maximum resection of DHGs.

Although the presence or absence of MI at the start of treatment can be a useful finding in predicting prognosis, even in MI-positive cases, maximal resection while preserving function, as in conventional GBM treatment, may improve the prognosis of DHG patients.

### Study limitations

This study is limited by the retrospective design and small sample size. Additionally, there were cases with no confirmed diagnosis, unreported cases, and cases with missing data including tumor size, which were not analyzed, resulting in a high degree of selection bias. Furthermore, the results may be biased because the effects of unmeasured confounders cannot be accounted for. Finally, image interpretation methods and surgical strategies varied between institutions. Future large-scale studies are needed.

## Conclusions

Similar to GBMs, DHGs have a poor prognosis if they invade deep structures or the contralateral cerebrum; however, safe and maximal tumor resection may prolong survival.

### Electronic supplementary material

Below is the link to the electronic supplementary material.

Supplementary Material 1

Supplementary Material 2

## Data Availability

No datasets were generated or analysed during the current study.
