# Supplementary figures and images for: Midline invasion predicts poor prognosis in diffuse hemispheric glioma, H3 G34-mutant: an individual participant data review
Source: J Neurooncol. 2024 Mar 1;167(1):201–10. doi: 10.1007/s11060-024-04587-5 (PMC10978637; doi:10.1007/s11060-024-04587-5)

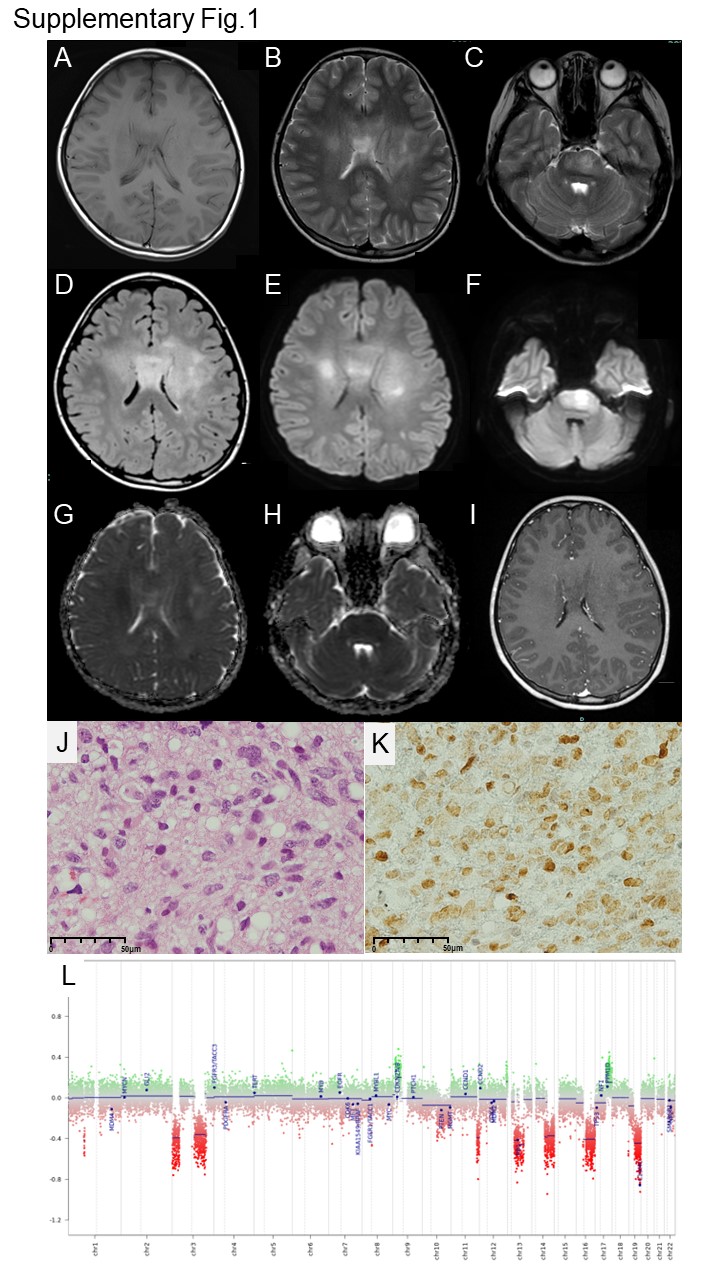

Supplement: Supplementary file 1 — Supplementary Material 1 [file 11060_2024_4587_MOESM1_ESM.jpg]
