# Supplementary material for: Midline invasion predicts poor prognosis in diffuse hemispheric glioma, H3 G34-mutant: an individual participant data review
Source: J Neurooncol. 2024 Mar 1;167(1):201–10. doi: 10.1007/s11060-024-04587-5 (PMC10978637; doi:10.1007/s11060-024-04587-5)
Supplement: Supplementary file 2 — Supplementary Material 2 [file 11060_2024_4587_MOESM2_ESM.docx]

Supplementary Table 1. Neurological findings in patients

| **Symptoms** | **N = (%)** |
| --- | --- |
| Headache | 23 (37.7) |
| Seizure | 20 (32.8) |
| Motor disorder | 14 (23.0) |
| Nausea | 14 (23.0) |
| Acute intracranial hypertension | 3 (4.9) |
| Memory disorder | 3 (4.9) |
| Sensory disorder | 3 (4.9) |
| Visual disturbance | 3 (4.9) |
| Disturbance of consciousness | 2 (3.3) |
| Dizziness / Vertigo | 2 (3.3) |
| Double vision / Oculomotor palsy | 2 (3.3) |
| Dysarthria | 2 (3.3) |
| Aphasia | 1 (1.6) |
| Facial palsy | 1 (1.6) |
| Facial twitching | 1 (1.6) |
| Urinary incontinence | 1 (1.6) |
| Missing data | 36 (37.1) |
